# Supplementary material for: Efficacy of an Enterotoxigenic Escherichia coli (ETEC) Vaccine on the Incidence and Severity of Traveler’s Diarrhea (TD): Evaluation of Alternative Endpoints and a TD Severity Score
Source: Microorganisms. 2023 Sep 27;11(10):2414. doi: 10.3390/microorganisms11102414 (PMC10609384; doi:10.3390/microorganisms11102414)
Supplement: Supplementary file 1 [file microorganisms-11-02414-s001.zip › microorganisms-2582206-supplementary.pdf]

Table S1. Trial Eligibility Criteria

**Parameter**	**Inclusion Criteria**
Age	≥18 years old at time of enrollment
Travel History	No travel to a developing country in the 12 months preceding study specific travel
Travel Plans	Established travel plans in or around Antigua, Guatemala, or Cuernavaca, Mexico during study period
General Health Status	Good general health as determined through physical exam
Preexisting Medical Conditions	Lack of gastrointestinal disease, or acute or chronic illness resulting in immunodeficiency as determined through medical history and physical exam
Pregnancy Status	Non-pregnant if female as confirmed through urine pregnancy test, post-menopausal status, or documentation of surgical sterilization.
Contraception Use	Willingness and ability to use a reliable contraceptive method during study enrollment period if female and able to conceive
Prophylactics	Willingness and ability to not use diarrheal prophylactics during study enrollment period outside of those provided through study clinic.
Antibiotics	Willingness and ability to not use antibiotics during study enrollment period outside of those provided through study clinic
Comprehension	Comprehension of study participant protocol as determined through a protocol comprehension test
Voluntary Participation	Willingness and ability to voluntarily participate as determined through signed consent obtained after informed consent counseling session
Counseling Session	Willingness and ability to participate in nurse supervised health counseling session conducted over the telephone
Dosing Session	Willingness and ability to participate in nurse supervised study product dosing session conducted over the telephone
Investigational Products	No prior use of any investigational or non-registered drug or vaccine other than the study vaccine(s) during the study period or within 30 days preceding the first dose of study vaccine
Prior Vaccination	No prior receipt of an oral cholera or ETEC vaccine with 5 years

Table S2. Diary Card Solicited Symptoms

Gastrointestinal Symptoms	General Symptoms
Decreased appetite	**Felt ill (malaise)**
Excessive Gas	Weakness
**Nausea**	Headache
**Vomiting**	Muscle ache
**Abdominal Pain/Cramps**	Chills
Gurgling stomach	Light-headedness
**Tenesmus**	Chills
Blood in stool	**Fever** (axillary/**oral temperature**)
**Unformed stool (maximum number/24 hrs)**	
Belching	
**Urgency of defecation**	

Table S3. Comparison of Gastrointestinal and General Symptoms Reported by Participants in the OEV-118 Trial Occurring During the 3 Days Following Receipt of the Inactivated Whole Cell ETEC Vaccine

	First Dose		Second Dose	
	Vaccine	Placebo	Vaccine	Placebo
No# of Participants	728	721	725	719
Abdominal pain	55 (7.5%)	53 (7.4%)	43 (5.9%)	40 (5.6%)
Abdominal cramps	77 (10.6%)	71 (9.9%)	54 (7.4%)	48 (6.7%)
Nausea	93 (12.8%)	64 (8.9%)	72 (9.9%)	56 (7.8%)
Vomiting[1]	11 (1.5%)	3 (0.4%)	20 (2.8%)	4 (0.6%)
Loss of appetite	48 (6.7%)	55 (7.6%)	40 (5.5%)	32 (4.5%)
Diarrhea[2]	4 (0.5%)	4 (0.6%)	8 (1.1%)	4 (0.6%)
Loose stools[3]	17 (2.3%)	24 (3.3%)	16 (2.2%)	16 (2.2%)
Feverishness	18 (2.5%)	14 (1.9%)	20 (2.8%)	13 (1.8%)

[1] Incidence of vomiting was significantly higher in vaccinees versus placebos after both dose 1 (p=0.03 by M-H) and dose 2 (p=0.001 by M-H). Incidence of diarrhea after the 2nd dose was not significantly different in the vaccine and placebo groups (p=0.25 by M-H).

[2] ≥3 unformed stools in 24 hrs

[3] ≥1 unformed stool in 24 hrs.

Note: There were two SAE's reported during the OEV-118 study; neither were judged to be related to the vaccine. Both occurred while subjects were at their travel destination and involved overnight hospitalization for treatment of Shigellosis (this subject was a vaccinee infected with *S. flexneri*) or dehydration associated with watery diarrhea attributed to ETEC infection (LT/ST+; CS1 + CS3 positive) (this subjects was a placebo recipient).
